# Humic substances increase tomato tolerance to osmotic stress while modulating vertically transmitted endophytic bacterial communities

**DOI:** 10.3389/fpls.2024.1488671

**Published:** 2024-11-19

**Authors:** Salomé Lengrand, Benjamin Dubois, Lena Pesenti, Frederic Debode, Anne Legrève

**Affiliations:** ^1^ Université catholique de Louvain (UCLouvain), Earth and Life Institute, Louvain-la-Neuve, Belgium; ^2^ Unit 1, Bioengineering, Walloon Agricultural Research Centre (CRA–W), Gembloux, Belgium

**Keywords:** humic acids, fulvic acids, drought stress, seed-borne endophytes, plant-endophyte interactions, endophytome composition, *Solanum lycopersicum*, hydroponics

## Abstract

While humic substances (HS) are recognized for their role in enhancing plant growth under abiotic stress by modulating hormonal and redox metabolisms, a key question remains: how do HS influence the microbiota associated with plants? This study hypothesizes that the effects of HS extend beyond plant physiology, impacting the plant-associated bacterial community. To explore this, we investigated the combined and individual impacts of HS and osmotic stress on tomato plant physiology and root endophytic communities. Tomatoes were grown within a sterile hydroponic system, which allowed the experiment to focus on seed-transmitted endophytic bacteria. Moreover, sequencing the 16S-ITS-23S region of the *rrn* operon (~4,500 bp) in a metabarcoding assay using the PNA-chr11 clamp nearly eliminated the reads assigned to *Solanum lycopersicum* and allowed the species-level identification of these communities. Our findings revealed that HS, osmotic stress, and their combined application induce changes in bacterial endophytic communities. Osmotic stress led to reduced plant growth and a decrease in *Bradyrhizobium* sp., while the application of HS under osmotic stress resulted in increased tomato growth, accompanied by an increase in *Frigoribacterium* sp., *Roseateles* sp., and *Hymenobacter* sp., along with a decrease in *Sphingomonas* sp. Finally, HS application under non-stress conditions did not affect plant growth but did alter the endophytic community, increasing *Hymenobacter* sp. and decreasing *Sphingomonas* sp. This study enhances the understanding of plant–endophyte interactions under stress and HS application, highlighting the significance of the vertically transmitted core microbiome in tomato roots and suggesting new insights into the mode of action of HS that was used as a biostimulant.

## 1 Introduction

Climate change poses a range of challenges to essential agricultural crops, including extreme weather events, temperature fluctuations, and, most notably, an increase in drought frequency (Zhou et al., 2024). Tomato (*S. lycopersicum*) is the most widely cultivated horticultural crop, produced at 186.1 million tons in 2022 (FAO, 2024; Hammond et al., 2022; Kopecká et al., 2023). However, drought stress severely impacts tomato production by disrupting key physiological processes, such as reducing photosynthesis, impairing water and nutrient uptake, and inducing oxidative stress through reactive oxygen species (ROS) accumulation (Li et al., 2024). These disruptions alter gene expression and metabolic pathways crucial to maintain osmotic balance, leading to stunted plant growth (Francesca et al., 2022; Landi et al., 2023). Consequently, tomato, which is particularly sensitive to water scarcity, experiences significant yield reductions, with losses reaching up to 40% (Hammond et al., 2022; Kopecká et al., 2023; Cruz-López et al., 2024).

To address this constraint, the use of biostimulants—substances or microbial inoculants that improve nutrient efficacy, stress tolerance, and crop quality—has gained attention (du Jardin, 2015). These sustainable products are increasingly important in mitigating the adverse effects of climate change, including the growing severity of droughts (du Jardin, 2015; Rouphael and Colla, 2018). Humic substances (HS), major components of soil organic matter resulting from the decomposition of plant, animal, and microbial residues, are one of the main categories of plant biostimulants (du Jardin, 2015). HS are a mixture of supramolecular conformations composed of relatively small and heterogeneous molecules associated by weak bonds (Piccolo, 2002; Mahler et al., 2021; Tiwari et al., 2023). They benefit plants indirectly by enhancing soil quality and its microbial community, providing carbon, improving soil aeration and stability, and facilitating nutrient uptake (Elkins and Nelson, 2002; Magdoff and Weil, 2004; Nardi et al., 2002; Piccolo, 2002). They also directly influence plant growth by activating hormonal pathways, particularly those of auxin, abscisic acid, and ethylene (Canellas et al., 2015). Some studies highlighted that HS contain auxins or have structural similarities with auxin and interact with hormone-cell receptors to initiate hormonal pathways (Canellas et al., 2002; Piccolo, 2002; Trevisan et al., 2010). Such activation leads to the stimulation of proton pumps, improving nutrient import within plants, increasing cell wall loosening and division, and enhancing root system development (Mora et al., 2010, 2012; Morsomme and Boutry, 2000; Muscolo et al., 2013; Nardi et al., 2002; Olaetxea et al., 2019; Quaggiotti, 2004; Trevisan et al., 2010; Zandonadi et al., 2007). The impact of HS on plants also comes from the activation of ROS pathways and the overexpression of antioxidant enzymes, which prime the plant without causing irreversible damage (Berbara and García, 2014; García et al., 2016; Schiavon et al., 2010). The beneficial effects of HS under water stress have been observed in various plants, including rice—where it helped protect cell membrane permeability—and *Brassica napus*, with reported increases in chlorophyll content, as well as in maize and tomato among others (Eyheraguibel et al., 2008; Galambos et al., 2020; Garcia et al., 2014; Lotfi et al., 2015). Despite in-depth studies on these products, our understanding of their biostimulation mechanisms remains incomplete. Their impacts are probably not only due to a direct influence on the plant but might also involve interactions with intermediary microorganisms (da Silva et al., 2021b).

Plants are known to harbor microbial communities within their tissues, known as endophytes, whose composition is shaped by various biotic and abiotic factors (Lengrand et al., 2024). These plant–microbe interactions are crucial for the adaptation and survival of both plants and microbes under stressful conditions (Meena et al., 2017; Meenakshi et al., 2019; Ullah et al., 2019)—for instance, bacterial endophytes can enhance plant growth through several mechanisms, such as improving nutrient uptake, producing hormones, and generating beneficial metabolites (Malinowski and Belesky, 2000; Ullah et al., 2019). Endophytes can be acquired from the environment or transmitted through seeds, with each group playing a vital role in plant health. Seed microbiota serve as a reservoir of microbial taxa that have co-evolved with plant hosts, providing functions that can support plant survival (Geisen et al., 2017; Hardoim et al., 2012; Johnston-Monje and Raizada, 2011). Moreover, this vertical transmission ensures the continuity of certain microorganisms from parent plants to their offspring, potentially promoting their beneficial growth. This process contributes to overall plant health and fosters beneficial endosymbiotic relationships (Barret et al., 2016; Cope-Selby et al., 2017; Hardoim et al., 2012; Rudgers et al., 2009; Shade et al., 2017).

Although HS and beneficial bacteria are effective as stand-alone biostimulants, numerous studies have shown that their positive impact on plant growth and stress tolerance is enhanced when used in co-inoculation (Canellas et al., 2013, 2015; da Silva et al., 2021c, da Silva et al., 2021a; Olivares et al., 2015)—for instance, Galambos et al. (2020) reported the combined use of HS with *Paraburkholderia phytofirmans* and *Pantoea agglomerans* that significantly promoted tomato growth, suggesting a complementary effect. This finding aligns with research by de Melo et al. (2018) and Olivares et al. (2015), which demonstrated improved plant vitality and stress tolerance when HS were used alongside microbial inoculants. In maize, Canellas et al. (2013) observed that HS application with *Herbaspirillum seropedicae* activated key metabolic pathways, enhancing the plant response to environmental challenges. Similarly, Baldotto et al. (2010) observed that such co-inoculation strategies led to significant improvements in nutrient uptake and overall plant health. The beneficial effects of co-inoculation of HS and plant-growth-promoting bacteria (PGPB) are likely due to multiple factors: (1) increased bacterial colonization and penetration (Nardi et al., 2009; Olivares et al., 2015, 2017; Piccolo, 2002; Canellas et al., 2008; Puglisi et al., 2008; Canellas et al., 2013), (2) activation of plant metabolic pathways that complement those induced by bacteria and HS individually (Galambos et al., 2020; Aguiar et al., 2016), and (3) direct effect of HS on bacterial metabolism (Wang et al., 2024). Since HS are known to influence co-inoculated bacteria, it stands to reason that they would similarly impact the communities of endophytic bacteria within plants.

In the present work, we hypothesize that HS positively influence plant growth and stress resilience concurrently with modifications in bacterial endophytic communities and that investigating these effects could help in understanding the mechanism of action of these molecules. To distinguish the direct effect of HS on the plant and their indirect effect via interactions with the soil microbiome, we developed a hydroponic system in sterile conditions. This setup allowed us to focus exclusively on seed-transmitted endophytic bacteria, avoiding any influence from the soil microbiome. This study analyzed the effects of HS on tomato growth under both non-stress and osmotic stress conditions, comparing the endophytic communities in roots across these different scenarios. The objectives were to determine (i) the impact of osmotic stress, (ii) the influence of HS, and (iii) the combined effects of HS and osmotic stress on both plant growth and the composition of the bacterial endophytic community. The results highlight the distinct effects of osmotic stress, HS, and their combined interaction on plant growth and the composition of the bacterial endophytic community.

## 2 Materials and methods

### 2.1 Impact of HS on plant growth

#### 2.1.1 Experimental design and tomato growth conditions

The experiment followed a completely randomized design. Four treatments were tested: (1) “Control”, (2) “PEG” for osmotic stress, (3) “HS” for humic substances, and (4) “PEG + HS” for HS application under osmotic stress. Six biological replicates were used per treatment, and all were harvested after 3 weeks. The experiment was repeated three times.

**Figure 1:**
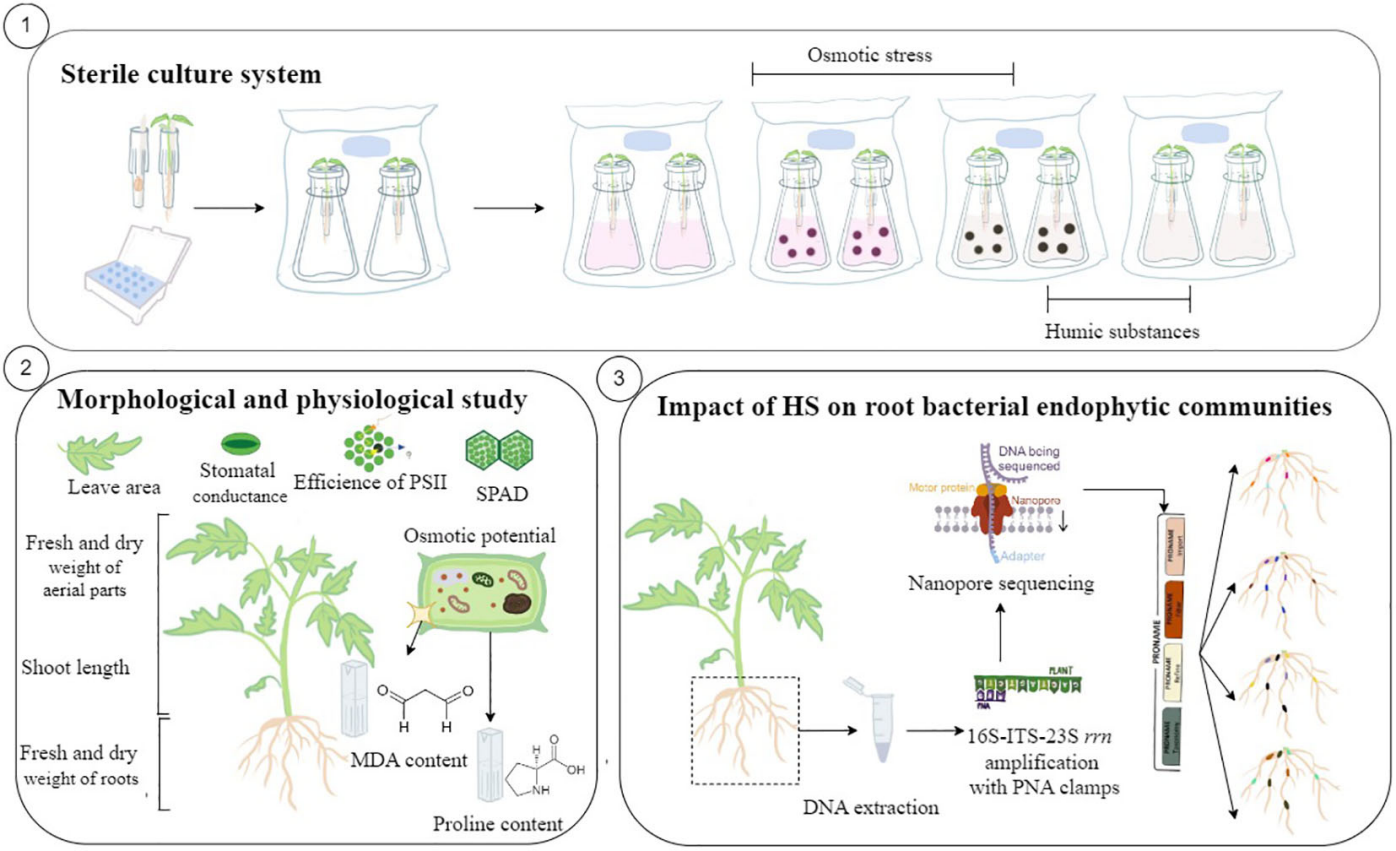
Schematic of the experimental workflow. (1) Under sterile conditions, a culture was made first in a germination system composed of a sterile box filled with sterile water and tips in which absorbent paper and seeds were placed. At the two-cotyledon stage, the seedlings were transferred into a custom-designed sterile hydroponic system filled with Hoagland’s solution adapted for tomato plants and enclosed in an autoclavable culture bag with a 0.02-µm filter to allow gas exchange. After 1 week of adaptation, both osmotic stress and humic substances were applied in the hydroponic solution. The plants were grown during 3 weeks, and the sterile hydroponic solution was replaced and the treatments were applied every week. (2) At harvest, the effects of HS, osmotic stress, and both were evaluated through morphological and physiological analyses including shoot length, fresh and dry weight of aerial parts and roots, leaf area, stomatal conductance, efficiency of photosystem II, SPAD, osmotic potential of leaves, and proline and MDA content in leaves. (3) The tomato plants grown under sterile conditions were harvested after 3 weeks, and DNA of the roots was extracted before 16S-ITS-23S rrn amplification (4,500 bp) with a new designed peptide nucleic acid clamp and Nanopore sequencing. Raw data were analyzed with PRONAME tools.

*S. lycopersicum* seeds (var. Moneymaker) commercially sourced from the Vilmorin gardening company (Saint-Quentin-Fallavier, France) were surface-disinfected in <5% sodium hypochlorite for 10 min under orbital shaking and washed three times in sterile distilled water (20 min). This concentration and timing of disinfection were selected to prevent any microbial growth on solid media post-disinfection while ensuring a high germination rate (over 95%). The seeds were germinated in a sterile box (70% relative humidity and temperature of 24°C/22°C (day/night)) for 10 days, 7 days in the dark and 3 days under light, to maintain optimal humidity for germination. The seedlings were then transferred into a custom-designed sterile hydroponic system which consisted of two Erlens (150 mL) filled with Hoagland’s solution adapted for *S. lycopersicum* (NH_4_NO_3_: 0.04 g · L^-1^; Ca(NO_3_)_2_ · 4H_2_O: 0.413 g · L^-1^; KNO_3_: 0.2035 g · L^-1^; KH_2_PO_4_: 0.137 g · L^-1^; MgSO_4_ · 7H_2_O: 0.123 g · L^-1^; MnSO_4_ · 5H_2_O: 0.265 mg · L^-1^; H_3_BO_3_: 0.7 mg · L^-1^; CuSO_4_ · 5H_2_O: 0.075 mg · L^-1^; (NH_4_)_6_Mo_7_O_24_ · 4H_2_O: 0.004 mg · L^-1^; ZnSO_4_ · 7H_2_O: 0.3 mg · L^-1^; Fe EDDHA: 0.03 g · L^-1^). This system was enclosed in an autoclavable culture bag with a 0.02-µm filter (Sun bag, transparent, B7026-100EA) to allow gas exchange, supported by a plastic frame and a sterile plant potholder, ensuring a controlled environment (**Figure 1**). The tomato plants were cultivated in a growth chamber under a 16/8-h (light/dark) photoperiod regime, 70% relative humidity, and temperature of 24°C/22°C (day/night). A detailed description and images of the germination and culture systems are provided in **Supplementary Figures S1** and **S2**.

For the osmotic stress treatment (“PEG”), the plants were cultivated in Hoagland’s solution supplemented with 10% w/v polyethylene glycol 6000 (PEG 6000), chosen based on preliminary tests to quickly induce stress without causing plant death. This adjustment shifted the osmotic potential from -0.02 to -0.2 MPa. Plants treated only with HS (“HS”) were grown in hydroponic solution supplemented with humic and fulvic acids at a concentration of 500 µL · L^-1^, which was determined as optimal in preliminary experiments (assessing the morphological effect of HS on tomato responses to osmotic stress). A third set (“PEG + HS”) received both PEG 6000 and humic and fulvic acids in their hydroponic solution. The control plants were grown in standard Hoagland’s solution. Both osmotic stress and HS were introduced one week after transferring the plants to the Erlenmeyers. The addition of HS, with or without PEG, did not significantly alter the osmotic potential of the solution (*p*-value >0.1) (**Supplementary Table S1**). PEG and HS were incorporated into the hydroponic solution prior to autoclaving, ensuring no contamination from either substance. The system was maintained for 3 weeks, and sterile hydroponic solutions were replenished every week under sterile conditions. This duration was chosen to allow the analysis of rapid effects on plant physiology and morphological responses without compromising plant viability. In parallel, control systems without plants were also maintained, and sterility was verified through DNA extraction and 16S rRNA amplification as well as plating the hydroponic solutions on culture media to ensure that no bacterial growth occurred.

The HS utilized in this study is the commercial biostimulant Humifirst^®^ from Tradecorp. This liquid formulation consists of 12% humic acids and 3% fulvic acids (w/w solution), extracted from leonardite. Its stable composition ensures reliable experimental outcomes, which is crucial for this type of research.

The content of Humifirst and its characteristics inferred from a chromatographic analysis have been meticulously calibrated and were previously detailed by Tahiri et al. (2016a, b) (**Supplementary Table S2**).

#### 2.1.2 Assessment of the morphological and physiological parameters of tomato plants

At harvest, the morphological and physiological variables were analyzed on six plants per treatment. The leaf area was scanned and analyzed using ImageJ. The stomatal conductance and the chlorophyll content were measured for each leaf at the four-leaf stage using the AP4 porometer and the SPAD chlorophyll meter, respectively. Measurements of stomatal conductance and relative chlorophyll content were taken at the center of each leaf, not on the vein. The efficiency of photosystem II was evaluated with a fluorimeter with two indicators: Fv/Fm which represents the maximum quantum yield of photosystem II and qPSII which is the actual quantum yield. The osmotic potential of leaves was measured with the VAPRO^®^ vapor pressure osmometer. The proline and malondialdehyde (MDA) contents of the leaves were quantified according to the protocol of Bates et al. (1973) and Heath and Packer (1968), respectively. Briefly, both protocols are based on a colorimetric determination measured with a spectrophotometer. The experiment has been reproduced three times. However, the proline and MDA determinations were only performed in one trial with six replicates per treatment.

### 2.2 Impact of osmotic stress, HS and combined effects on endophytic communities

#### 2.2.1 Plant growth and DNA extraction

During the third and final assay of HS application on tomato grown under non-stress and osmotic stress conditions, impact on endophytic communities was evaluated. At harvest, five plants per treatment (Control, HS, PEG + HS, and PEG) were surface-disinfected. Briefly, entire tomato plants were first placed in NAP buffer (124 mM Na_2_HPO_4_) and sonicated in an ultrasonic cleaning bath (VWR^®^) for 1 min to dislodge bacteria on the surface. The plants were then submerged in sodium hypochlorite solution (5.25%) for 6 min and rinsed with sterile water (Ruiz-Perez and Zambrano, 2017). The intact plants were dried on sterile paper. Sterilization was checked by making an imprint of the plants on Luria broth agar (LBA), and the plates were checked for 2 weeks (Fitzpatrick et al., 2018b). Disinfection was carried out on all plants to avoid the disinfectant solution from penetrating the interior of the plants. Only the roots were kept for the endophytic communities to be studied, based on the results of da Silva et al. (2021b) and De Hita et al. (2020). Each root system was ground separately in liquid nitrogen under sterile conditions, and DNA was extracted from 50 mg of ground tissue using a modified CTAB-based method. Briefly, NaCl (100 µL, 5 M) was added before adding isoamyl alcohol/chloroform to reduce the high polysaccharide concentration in the extractant (Fang et al., 1992). DNA pellet was diluted in 20 µL of nuclease-free water.

#### 2.2.2 PCR amplification of a ribosomal marker of endophytic bacteria with three PNA clamps

The extracted DNA was amplified using the primers 16S-8F (5′-AGRGTTYGATYMTGGCTCAG-3′) and 23S-2490R (5′-CGACATCGAGGTGCCAAAC-3′), targeting the 16S-ITS-23S region of the ribosomal operon (*rrn*) (~4,500 bp) of bacteria (Karst et al., 2021). The universality of these primers was tested by comparing the genera amplified with three commonly used primer pairs (Lengrand et al., manuscript submitted for publication). The PCR mixture (25 µL total volume) contained 2×GoTaq Long PCR Master Mix, 0.4 µM of each primer, 2 µM of mPNA (to block mitochondrial amplification), 2 µM of pPNA (to block plastid amplification) (Lundberg et al., 2013), 2 µM of PNA-chr11 (CTGCTAATACCYCGKAGGCTGA) (to block chromosome 11 amplification of *S. lycopersicum* var. Moneymaker) (Lengrand et al., manuscript submitted for publication), and 20 ng of DNA. The PCR thermal program consisted of initial denaturation of 2 min at 95°C, followed by 30 cycles of 20 s at 95°C, 60 s at 74°C, 30 s at 55°C, 5 min at 72°C, and a final extension of 10 min at 72°C. GoTaq Long PCR Master Mix was chosen for its ability to work in the presence of HS (Matheson et al., 2014) since they are inhibitors of PCR (Sidstedt et al., 2015). For each sample, PCR was performed in three replicates and pooled before purification to assure homogeneity. The amplicons of the 16S-ITS-23S region of the *rrn* operon were cleaned up with AMPure XP beads (Beckman Coulter) using a 0.5X bead-to-sample ratio. Quantity and quality were evaluated using Quantus™ Fluorometer (Promega) and a Nanodrop spectrophotometer (ThermoFisher Scientific), respectively. The PCR products were visualized on 0.8% agarose gel.

#### 2.2.3 Library preparation

Libraries were prepared using Ligation Sequencing Kit V14 (SQK-LSK114) (Oxford Nanopore Technologies, Oxford, UK) according to the manufacturer’s instructions. Briefly, amplicons were processed for end repair using NEBNextUltra II End Repair/dA-tailing Module (New England Biolabs, Ipswich, MA, USA), and sequencing adapters were attached. The libraries were sequenced with a MinION device (Oxford Nanopore Technologies) using Flongle Flow Cells (R10.4.1) for 24 h.

#### 2.2.4 Sequence data processing

Raw data were analyzed with PRONAME (Dubois et al., manuscript submitted for publication). Briefly, raw data were imported into PRONAME, adapters and primers were trimmed, and a graph of simplex and duplex read distribution was constructed. Data were filtered by keeping only duplex reads with a length between 3,500 and 5,000 bp and with a minimum quality score of 15. PRONAME clustered reads with a percentage of identity of 90%, centroid sequences were polished using Medaka, and chimera sequences were discarded. The taxonomic analysis of representative sequences was carried out using the blastn standalone tool (v2.15.0) (Camacho et al., 2009) from BLAST command line applications and the curated rEGEN-B database included in the PRONAME pipeline and dedicated to bacterial 16S-ITS-23S *rrn* operon region. Composition analysis of data was done using the phyloseq package (McMurdie and Holmes, 2013) in RStudio, and results were visualized with the ggplot2 package (Wickham, 2014).

### 2.3 Statistical analysis

The impact of HS on tomato growth was evaluated three times, and data were analyzed with RStudio. After validating the data for normal distribution (Shapiro–Wilk test, *p*-value >0.05) and variance homogeneity, each experiment was analyzed individually, and two-way analysis of variance (ANOVA) was used to demonstrate non-significant differences between the three trials (*p*-value >0.05). Data from the three experiments were pooled, and significant differences among treatments were assessed with Student’s *t*-test (*p* ≤ 0.05). As the stomatal conductance and the relative chlorophyll content were measured on each leaf of the plants, the results were analyzed according to a mixed model with the “plant” parameter.

The composition of endophytic communities associated with the roots was analyzed during the third repetition. Differences in bacterial endophytic communities according to the treatment applied (Control, PEG, HS, and PEG + HS) were evaluated using both alpha and beta diversity. Alpha diversity was calculated using the vegan R package (Oksanen et al., 2009) with observed features, Shannon entropy, and Pielou evenness, followed by Kruskal–Wallis test. Beta diversity was analyzed by principal coordinates analysis (PCoA) using the ape package (Paradis, 2024) and by performing an analysis of similarity (ANOSIM) test using the vegan package (Oksanen et al., 2009). Beta diversity was analyzed at the species and genera levels. Finally, identification of taxonomic groups with statistically significant differences in abundance between treatments was performed using ANCOM-BC (Lin et al., 2022) on QIIME2 v2024.2 (Bolyen et al., 2019). This method acknowledges sources of variation in microbiome datasets, including unequal sampling fractions and differences in sequencing efficiency, to attempt to limit the effect of group unevenness due to the microbiome variability among individuals in our study. The operational taxonomic units (OTU) were summarized at the genus level.

## 3 Results

### 3.1 Impact of HS on plant growth in non-stress and osmotic stress conditions

**Figure 2:**
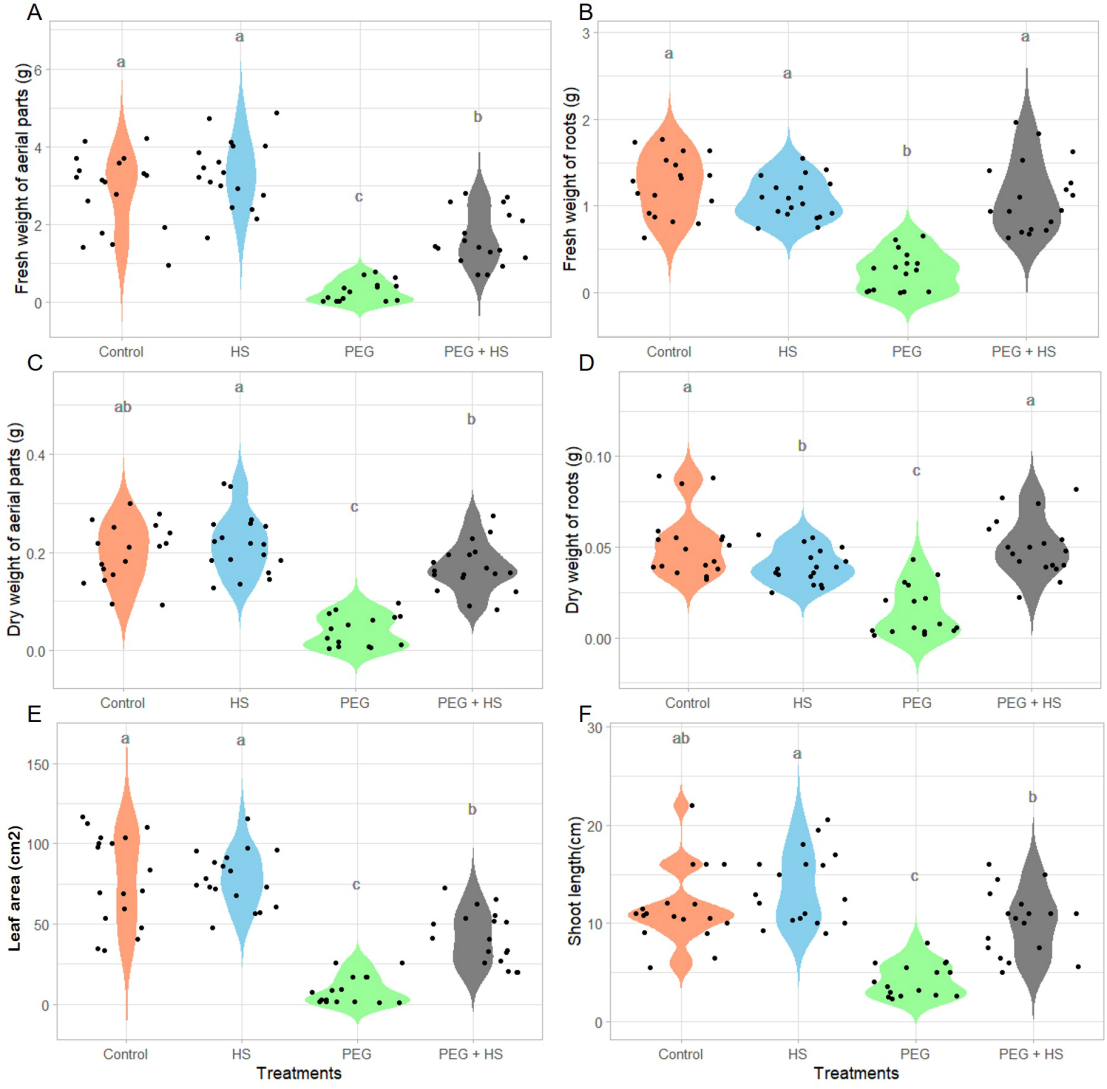
Impact of osmotic stress and humic substances on the morphological traits of tomato. Fresh and dry weight of aerial parts **(A, C)** and roots **(B, D)**, leaf area **(E)**, and shoot length **(F)** of untreated plants (Control), non-stress condition and treated with humic substances (HS), plants under osmotic stress (PEG), and plants under osmotic stress and treated with HS (PEG + HS) after 3 weeks of treatment. Multiple comparisons were done with the Wilcoxon test (*p*-value <0.05). *N* = 18. Different lowercase letters indicate significant differences between treatments according to Wilcoxon test (p-value <0.05).

**Figure 3:**
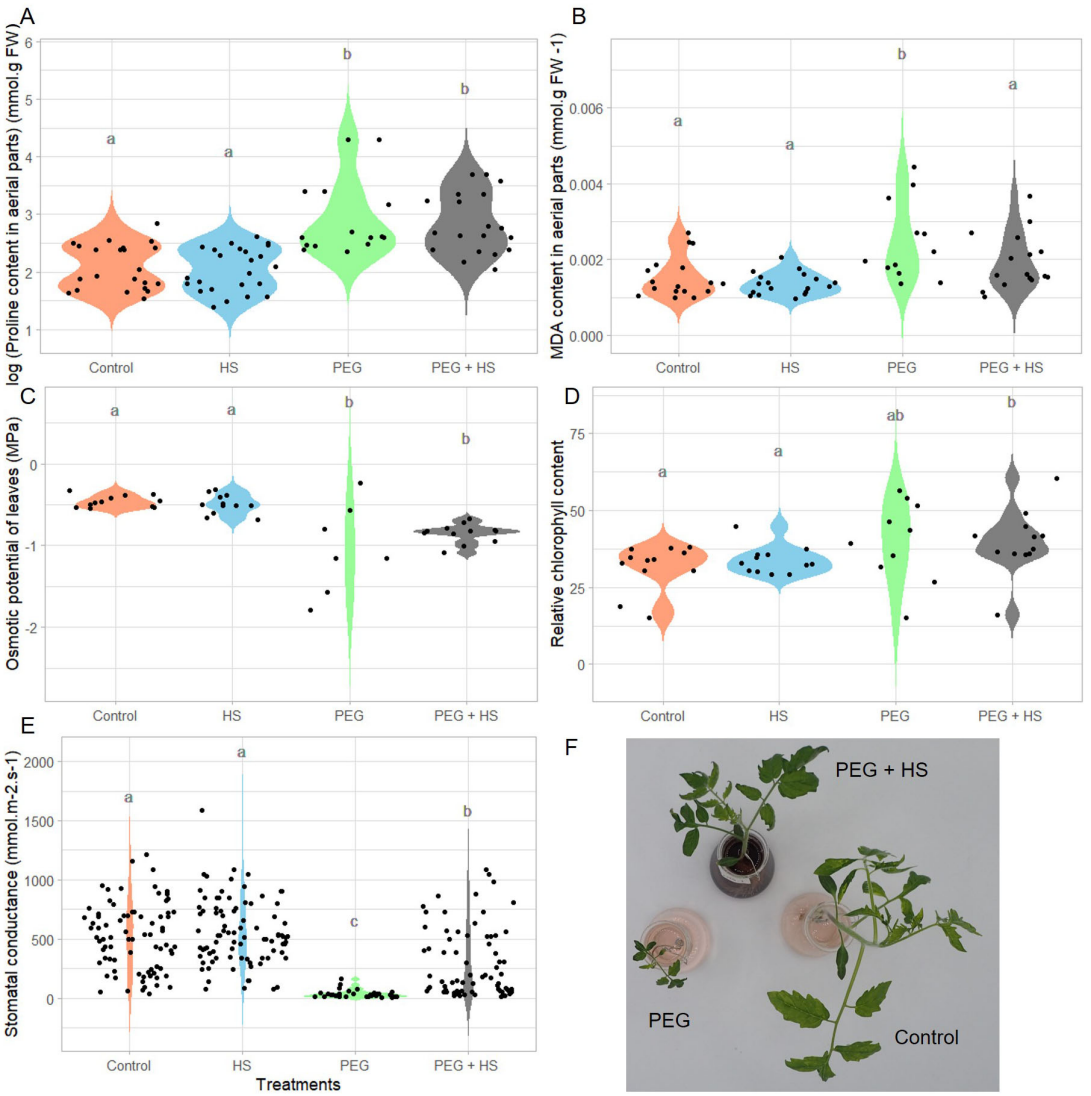
Impact of osmotic stress and humic substances on the physiological traits of tomato. Proline and malondialdehyde (MDA) content in aerial parts **(A, B)**, osmotic potential and relative chlorophyll content of leaves **(C, D)**, and leaf stomatal conductance **(E)** of untreated plants (Control), non-stress condition and treated with humic substances (HS), plants under osmotic stress (PEG), and plants under osmotic stress and treated with HS (PEG + HS) after 3 weeks of treatment. Images of plants treated with PEG, PEG + HS and control are shown in **(F)**. Multiple comparisons were done with the Wilcoxon test (*p*-value <0.05). *N* = 18. Different lowercase letters indicate significant differences between treatments according to Wilcoxon test (p-value <0.05).

In the absence of stress, HS application did not significantly affect most of the measured growth parameters (**Figure 2**, **3**). A minor reduction in the dry weight of roots was noted in comparison to “Control” plants, without a noticeable difference in the fresh weight of roots (**Figures 2B, D**). In contrast, a slight but not significant enhancement in the fresh weight of the aerial parts was observed (**Figure 2A**).

Plants grown under polyethylene glycol (“PEG”)-induced osmotic stress showed a general decrease in growth. Indeed reduced fresh and dry weights, shoot length, and leaf area were observed after 3 weeks of treatment (**Figure 2**). In parallel, the tomato plants showed an accumulation of proline in the aerial parts (**Figure 3A**) and stomatal closure (**Figure 3E**) combined with a reduced osmotic potential of leaves (**Figure 3C**). Parallel with these modifications, the plants were subjected to oxidative stress, as evidenced by an increase in lipid membrane peroxidation resulting in elevated MDA content in the aerial parts (**Figure 3B**). Osmotic stress resulted in no significant alterations in the relative chlorophyll content (**Figure 3D**) and in the efficiency of photosystem II (**Supplementary Table S3**).

Under osmotic stress, the addition of HS significantly enhanced the aerial part and root fresh weights (484% and 339%, respectively) as well as the aerial part and root dry weights which showed increases of 324% and 239%, respectively (**Figures 2A–D**). These increases were observed parallel with leaf area enlargement (**Figure 2E**) and an increase in shoot length (**Figure 2F**). Moreover, plants treated with HS under osmotic stress showed a reduced MDA content (**Figure 3B**) and increased stomatal conductance (**Figure 3E**) compared to plants under osmotic stress. Finally, both “PEG” and “PEG + HS” plants exhibited an increase in proline content and a similar reduction in osmotic potential (**Figures 3A, C**).

### 3.2 Shift induced by osmotic stress and humic substances in endophytic bacterial community composition

The metabarcoding Nanopore sequencing of 20 samples resulted in 613,942 high-quality duplex reads. One sample from the HS treatment was excluded due to an insufficient number of reads, resulting in four samples for this treatment. The bioinformatic reconstruction of root endophytic communities identified 59 distinct representative sequences classified into five phyla. For reasons of simplicity, we will call them OTUs. Proteobacteria emerged as the most predominant phylum across all conditions, with relative abundances of 99.84%, 75.33%, 99.33%, and 84.02% in the “Control”, “HS”, “PEG”, and “PEG + HS” treatments, respectively.

**Figure 4:**
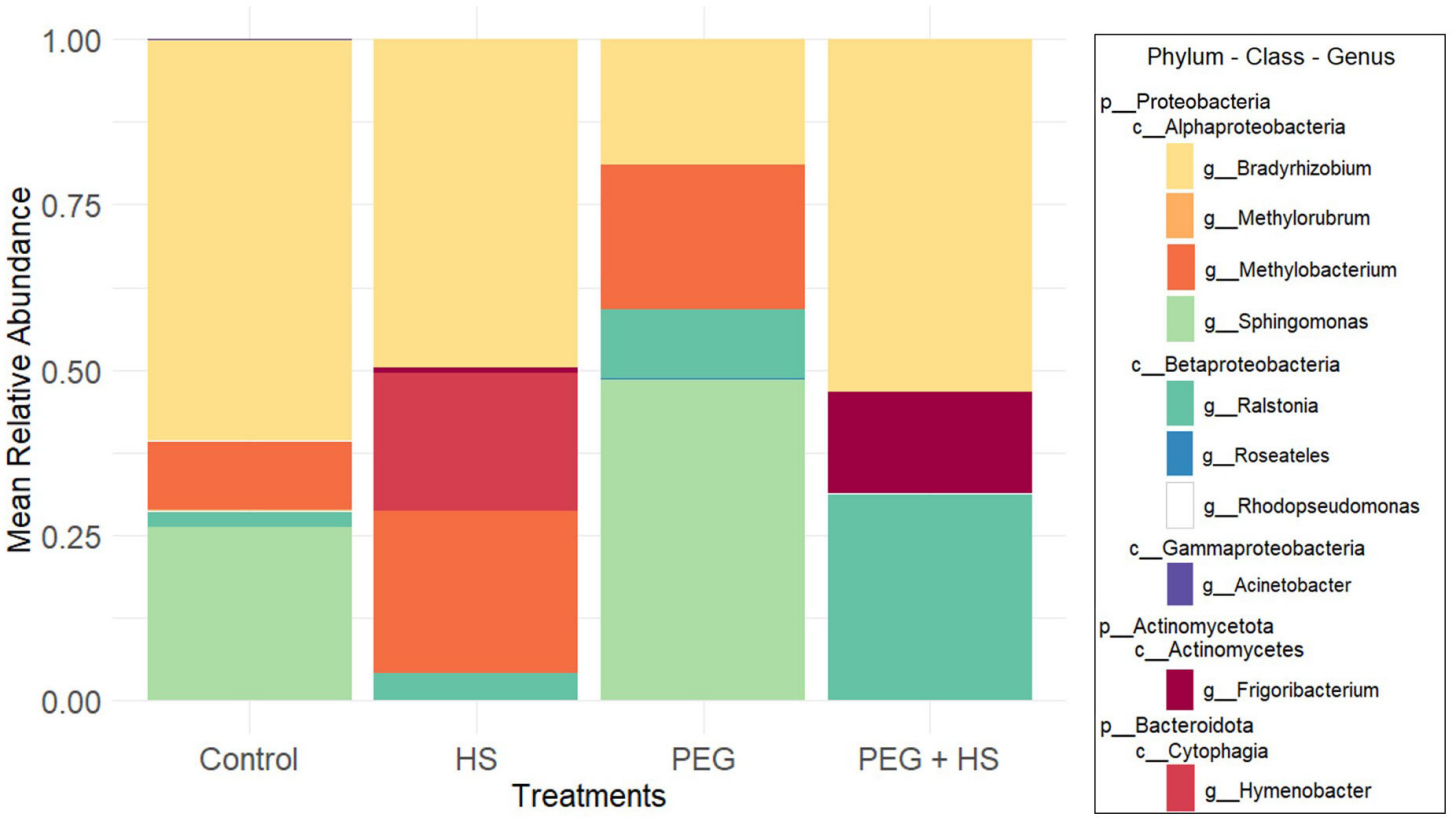
Mean relative abundance of endophytic bacterial taxa, at the genus level, in the roots of tomato plants according to the treatment applied. “Control”, “HS”, “PEG”, and “PEG + HS” stand for untreated plants, no stress and treated with humic substances (HS), osmotic stress, and osmotic stress and treated with HS, respectively (*n* = 6).

The genera with high relative abundance were similar in the “Control” and “PEG”-treated groups, comprising *Bradyrhizobium*, *Methylobacterium*, *Ralstonia*, and *Sphingomonas*. In the HS treatment, *Frigoribacterium* and *Hymenobacter* appeared in high abundance alongside *Bradyrhizobium*, *Methylobacterium*, and *Ralstonia*. Finally, the combined PEG + HS treatment reduced the major genera to *Bradyrhizobium*, *Frigoribacterium*, and *Ralstonia*. *Bradyrhizobium* was predominantly found in all treatments except under PEG conditions, where *Sphingomonas* accounted for nearly half of the observed sequences (**Figure 4**).

Bacterial richness was consistent between “Control” and “HS”-treated plants. However, following osmotic stress treatment, a significant decrease was observed in bacterial richness in both “PEG”- and “PEG + HS”-treated plants, with a significant difference in alpha diversity analyzed with Shannon entropy and Pielou evenness (**Supplementary Figures S3**-**S5**).

Distinct patterns emerged in the beta diversity among different treatments, as illustrated by the principal coordinates analysis (PCoA; **Supplementary Figure S6**). Despite some intra-treatment variability observed in the samples (**Supplementary Figure S7**), ANOSIM analysis confirmed significant differences in the beta diversity of the endophytic community across the four treatments at both the genus level (*p*-value = 0.0349, *R* ^2^ = 0.30689) and the species level (*p*-value = 0.0297, *R* ^2^ = 0.29165). These variations were significantly influenced by the application of HS (as detailed in **Table 1**).

**Table 1:** Analysis of similarity of the impact of both osmotic stress and/or HS on bacterial endophytic communities in the roots of tomato plants at the genus and species levels.

	Genus		Species	
	*p*-value	$R^{2}$	*p*-value	$R^{2}$
Treatment	0.0349*	0.30689	0.0315*	0.26163
HS application	0.0084**	0.16202	0.0137*	0.1369
PEG application	0.2420	0.0635	0.1511	0.749

The treatment comprised both humic substances (HS) and polyethylene glycol (PEG) application.

*p-value <0.05; **0.01 < *p*-value <0.05.

**Figure 5:**
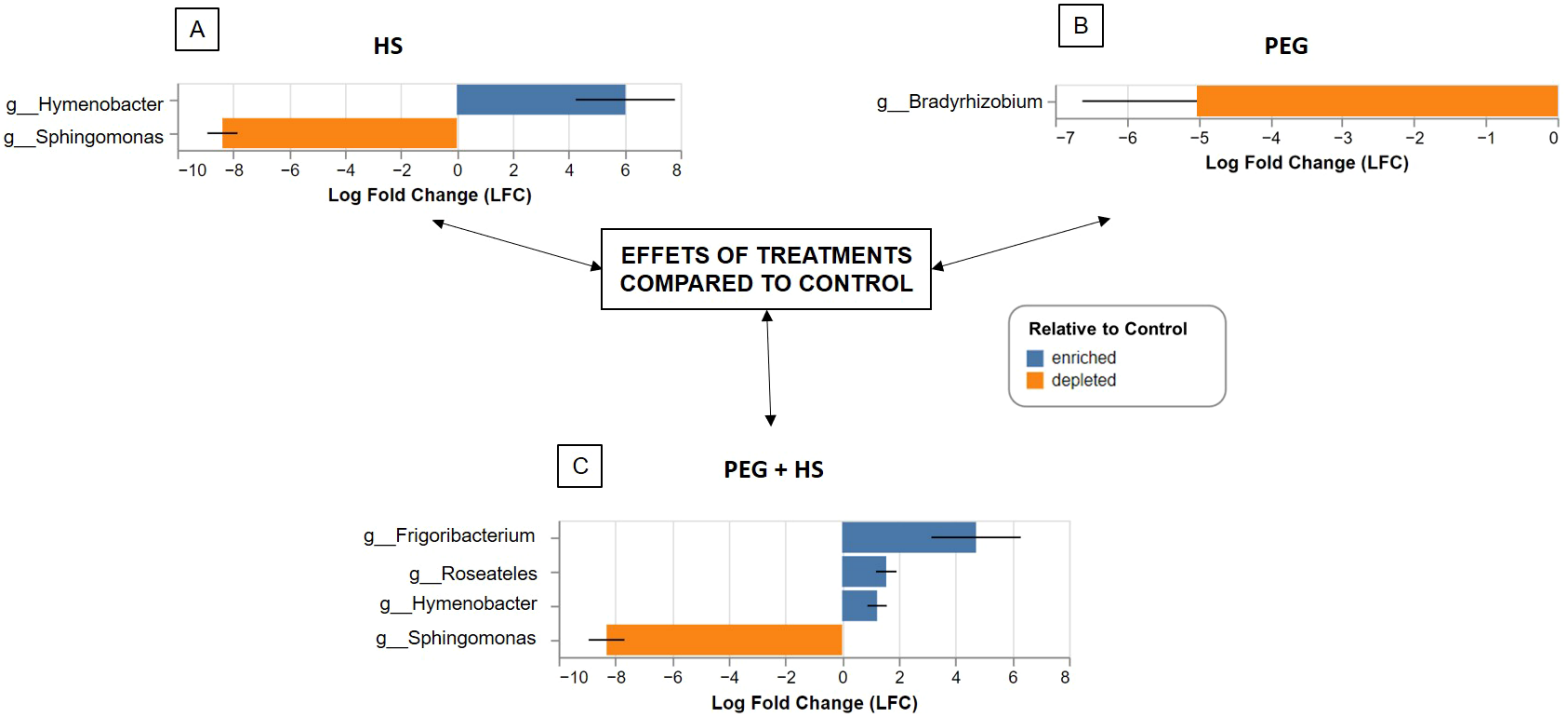
Bacterial genera enriched or depleted in tomato roots treated with humic substances (HS) **(A)**, under osmotic stress **(B)**, and under osmotic stress and treated with HS **(C)** compared to control plants. Significant differences in the relative abundance of taxa at the genus level were inferred using the analysis of compositions of microbiomes with bias correction (ANCOM-BC) tool.

Notably, applying the biostimulant without osmotic stress led to an increase in *Hymenobacter* genus (Bacteroidota phylum) and a reduction in Proteobacteria levels by decreasing the *Sphingomonas* genus (**Figure 4**). This observation was confirmed by the ANCOM-BC results (**Figure 5**). At the species level, there was an enrichment of reads assigned to *Hymenobacter* sp. BRD128 and a reduction of *Sphingomonas aerolata*.

Combining HS and osmotic stress induced an increase in the genera *Frigoribacterium*, *Roseateles*, and *Hymenobacter* as well as a decrease in the abundance of *Sphingomonas* compared to “Control” plants (**Figures 4**, **5**).

Finally, osmotic stress application did not significantly alter the beta diversity (**Table 1**) but did induce a reduction in *Bradyrhizobium* genus abundance (**Figures 4**, **5B**).

## 4 Discussion

This study employed a sterile hydroponic system to isolate the direct effects of HS on plant physiology from interactions with soil microbes. In this particular system, the results indicate that, under osmotic stress, HS application enhanced tomato resilience, evidenced by increased shoot and root weights, leaf area, shoot length, total chlorophyll content, and stomatal conductance. This growth enhancement is likely due to HS’ protective effects, activating antioxidant enzymes and thereby reducing oxidative stress, as indicated by the lower MDA content in leaves. The observed decrease in MDA levels post-HS application points to reduced lipid membrane peroxidation, a marker of oxidative damage. This aligns with other studies showing that HS application under stress conditions leads to a healthier plant state through enhanced antioxidant enzyme activities such as those of superoxide dismutase (SOD), catalase (CAT), ascorbate peroxidase (APX), and peroxidase (POX) (Aguiar et al., 2016; García et al., 2012; Lotfi et al., 2015; Matuszak-Slamani et al., 2022)—for example, in the experiment of Matuszak-Slamani et al. (2022) on soybeans under osmotic stress in hydroponics, humic acid (HA) application led to an increase in plant length and chlorophyll content parallel with an upregulation of the antioxidant defense system. Similar results were observed in sugarcane treated with HA under water stress, which displayed better growth than control plants with increased stomatal conductance, transpiration, and net photosynthesis. This induced tolerance was also correlated with the activation of antioxidant enzymes’ activities (Aguiar et al., 2016). On the contrary, HS application did not significantly affect tomato growth under non-stress conditions. This result aligns with existing literature in hydroponics showing that HS-containing biostimulants have limited effects in unstressed environments (Aguiar et al., 2016; García et al., 2012; Lüdtke et al., 2021; Matuszak-Slamani et al., 2022). The effects of HS as biostimulants are highly dependent on factors such as dosage, plant species, and the experimental system used (Jindo et al., 2020). In this case, the concentration selected was optimized to demonstrate the beneficial effects of HS under osmotic stress.

The result of this experiment revealed that the increase of tomato resilience to osmotic stress is associated with its direct interaction with plants or its phytobiome and not with an indirect effect through the soil. Since seed-transmitted endophytic communities of plants are now well recognized to play a role in plant health (Pandey et al., 2022; War et al., 2023), we deepened the taxonomic characterization of these communities by PCR amplification and sequencing from total DNA extracted from disinfected roots. The use of PNA-chr11, tailored specifically to reduce *S. lycopersicum* var. Moneymaker amplification (Lengrand et al., manuscript submitted for publication), and primers targeting the 16S-ITS-23S *rrn* operon enabled us to achieve species-level identification of endophytes. The primary microbiota present in the roots and transmitted through seeds were identified as *Bradyrhizobium*, *Methylobacterium*, *Ralstonia*, *Frigoribacterium*, *Hymenobacter*, and *Sphingomonas*, with specific reads assigned to *B.* sp. *SK17*, *M. oryzae*, *M.* sp. *FF17*, *M.* sp. *NMS14P*, *R. pickettii*, *F.* sp. *NBH87*, *H.* sp. *BRD128*, and *S. aerolata*. This finding aligns with the results of Lengrand et al. (manuscript submitted for publication) which identified the core microbiome of *S. lycopersicum* var. Moneymaker transmitted from seed to shoots and roots as *R. pickettii*, *S. aerolata*, *B.* sp. *SK17*, and *P. aquatile*. Additionally, the genera *Frigoribacterium* and *Hymenobacter* were also found in the leaves and roots of plantlets grown in sterile conditions.

The composition of the endophytic microbiome in tomato plants is highly sensitive to environmental conditions. In this study, the tomatoes were grown under sterile conditions, and the hydroponic solution containing PEG, HS, or both was autoclaved before use. Therefore, the observed shifts in the microbiome are not attributable to external microorganisms. The sterile conditions likely reduced the diversity of the endophytic communities by preventing the introduction of soil-derived microbes.

Notably, osmotic stress alters bacterial community composition by reducing the alpha diversity in both HS-treated and untreated plants. A similar reduction of bacterial richness in the roots of *Atractylodes lancea* grown under PEG-induced osmotic stress was observed in the experiment of Wang et al. (2022). Additionally, the application of PEG alone induced a notable shift in the root endophytic community composition, specifically reducing the prevalence of the *Bradyrhizobium* genus. This reduction may be due to the greater resilience of genera like *Sphingomonas* and *Ralstonia* under stress conditions, which could outcompete *Bradyrhizobium* for resources in stressful environments.

Under both stress and non-stress conditions, the application of HS led to an increase of Bacteroidetes, especially of *Hymenobacter*, with a reduction in Proteobacteria—with an almost complete suppression of *Sphingomonas*. Interestingly, the same conclusion was drawn in the experiment of da Silva et al. (2021b) in rice root endophytic communities, suggesting a consistent effect across different plant species. This influence of HS on bacterial communities seems to extend beyond plant-associated microbiota in the literature, as a similar shift after HS application was also observed in wastewaters and lakes (Hutalle-Schmelzer and Grossart, 2009; Luo et al., 2019). Indeed in ammonium-rich wastewater from landfill leachate, HS application diminished Proteobacteria while enhancing Bacteroidetes with a significant change for aerobic ammonium-oxidizing bacteria (AOB), crucial for nitritation-anammox processes (Luo et al., 2019). Similar adjustments were also observed in the microbiota of oligotrophic Lake Stechlin, where HS addition specifically enriched groups within Alphaproteobacteria, Bacteroidetes, and Deltaproteobacteria, indicating a direct HS effect on microbiota rather than an indirect plant-mediated one (Hutalle-Schmelzer and Grossart, 2009). The interaction between HS and microbial communities may be explained in part by the degradation of HS by bacteria. Under anaerobic conditions, bacteria involved in the anaerobic ammonium oxidation (Anammox) process contribute to HS degradation (Liang et al., 2009). Indeed reduced HS can act as electron donors for anaerobic organisms utilizing alternative electron acceptors, allowing these microorganisms to derive energy from HS by using carbon sources as acetate (Liang et al., 2009; Lipczynska-Kochany, 2018). This hypothesis might account for the observed shift, particularly for *Hymenobacter*, the predominantly favored genus, which includes numerous species known to function as ammonia-oxidizing bacteria (Garcia-Lopez et al., 2019; Zhang et al., 2020). In addition, HS contain a high concentration of dissolved organic carbon (DOC) (**Supplementary Table S2**), which can contribute to microbial nutrition (Tahiri et al., 2016a, b). Apart from DOC, the other nutrients in HS should be negligible in plant or microbial nutrition compared with the nutrients present in the hydroponic solution since they represent, for those present in larger quantities (N and Fe), concentrations a thousand times lower.

In parallel, Actinobacteria have also been found to be active in degrading HS in freshwater and estuarine environments (Haukka et al., 2005; Hutalle-Schmelzer and Grossart, 2009; Rocker et al., 2012; Lipczynska-Kochany, 2018). However, an increase in the Actinobacteria phylum, particularly the *Frigoribacterium* genus, was only observed in plants under osmotic stress that were treated with HS. This increase was notably absent when either the biostimulant or the osmotic stress was applied alone. Interestingly, an increase in Actinobacteria within the root microbiome has already been correlated with enhanced drought resilience in plants (Fitzpatrick et al., 2018a; Martins et al., 2023; Naylor et al., 2017; Santos-Medellín et al., 2017; Schimel et al., 2007; Tocheva et al., 2016). Xu et al. (2021) demonstrated that Actinobacteria enrichment in drought stress conditions may be partially due to a reduction of iron homeostasis (increase in insoluble Fe^3+^ in dried soils and reduction of plant Fe transporters) within the root under drought stress. Indeed the root’s reduced iron uptake and increased iron storage lead to low available iron concentrations in the root, benefiting bacteria proficient in scavenging the limited iron and to the detriment of less capable ones. This hypothesis gained support from the observation of Xu et al. (2021), where external application of iron interrupts the enrichment of Actinobacteria and, in parallel, reduces the drought stress tolerance of sorghum. In our experiment, osmotic stress induced in hydroponic systems might not affect Fe^3+^ availability, but HS could create soluble nutrient complexes (with Fe and phosphate among others) absorbable by plants (Spark et al., 1997). However, this absorption mainly involves fulvic acids and not larger humic acid molecules that only interact with cell walls and cannot be absorbed (Nardi et al., 2002). Consequently, the formation of such complexes with Fe, coupled with diminished water uptake due to osmotic stress, could mimic a drought stress effect on reduced Fe availability and increase Actinobacteria’s relative abundance. The increased abundance of Actinobacteria in line with higher plant drought tolerance is reported in numerous studies (Ebrahimi-Zarandi et al., 2023). A variety of mechanisms are used by these bacteria, including increasing nutrient availability via phosphate and potassium solubilization, siderophore production, and nitrogen fixation as well as the production of ACC deaminase, phytohormones, volatile organic compounds, extracellular polysaccharides, and osmolytes (Boukhatem et al., 2022). They also activate the plant’s antioxidant system and induce the expression of stress-responsive genes. A meta-transcriptomic analysis conducted by Xu et al. (2018) on sorghum roots under drought stress revealed significant alterations in actinobacterial gene expression across different functional categories, highlighting the critical roles of carbohydrates, amino acid transport, and ATP-binding cassette (ABC) transporters. The *Frigoribacterium* genera, representing the major increase of Actinobacteria in this study, were already known to solubilize phosphate and to produce siderophores with ACC deaminase activity (Boukhatem et al., 2022; Ebrahimi-Zarandi et al., 2023) as exemplified by the strain *F. faeni* 801, isolated from halophytes and with high tolerance to salt stress (Zhou et al., 2017).

Finally, the genus *Roseateles*, whose abundance was increased under combined osmotic and HS treatment, encompasses species known for their remarkable degradation capabilities. Among these, the strain *R. depolymerans* KCTC42856 stands out for its ability to break down aliphatic polymers in biodegradable plastics (Lee et al., 2016). Similarly, *R. chitinivorans* HWN-4^T^ has been identified as a promising candidate for bioremediation, capable of mitigating environmental contaminants (Sisinthy and Gundlapally, 2020). In the experiment of Le et al. (2023), further insights from antiSMASH analysis highlighted the significant variability in secondary metabolite profiles across *Roseateles* strains, suggesting a rich repertoire of defense mechanisms against competitive microbial species (Le et al., 2023). Considering the characteristics of this genus, it was probably more resilient to osmotic stress induced by PEG and had the ability to surpass competing bacteria by producing certain metabolites to gain access to limited resources.

In summary, endophytic communities carried by tomato seeds and transmitted to the roots were distinctly influenced by osmotic stress and the application of HS, either individually or in combination. The variations following the application of HS can be attributed to several hypotheses: (1) the composition of the HS may favor certain genera capable of using these compounds, indicating a direct nutritional impact, specifically for the *Hymenobacter* genus (Liang et al., 2009; Lipczynska-Kochany, 2018; Luo et al., 2019). Additionally, the effect of HS might be mediated by the release of metabolites, which could influence microbial community dynamics by promoting specific microbial taxa or altering interactions within the community; (2) Indirectly, HS could modify plant metabolism such as root iron homeostasis and favor specific phyla such as Actinobacteria with the *Frigoribacterium* genus (Fitzpatrick et al., 2018a; Nardi et al., 2002; Xu et al., 2021); (3) The recruitment of certain microbes could respond to HS-induced mild stress, activating plant defense mechanisms (Berbara and García, 2014; da Silva et al., 2021b; García et al., 2016); (4) The effects of HS could also be independent of endophytic composition, leading to a parallel benefit for plants and a change in their microbiota communities (Berbara and García, 2014; da Silva et al., 2021b; García et al., 2016).

In conclusion, this study investigated the combined and individual impacts of HS and osmotic stress on plant physiology and endophytic communities, revealing a possible correlation between endophytic communities and plant physiology, even in sterile environments. This underscores the importance of seed-borne bacteria as a primary inoculum. Moreover, HS application under osmotic stress increased tomato growth parameters in concert with the abundance of *Frigoribacterium* sp., *Roseateles* sp., and *Hymenobacter* sp. and reduction of *Sphingomonas* sp., highlighting the potential of HS to enhance certain plant–endophyte relationships. This opens new perspectives on the mode of action of HS, possibly by interacting with endophytes. Based on the findings of this study, the application of HS in agriculture and hydroponic systems should be carefully tailored to maximize both plant health and the beneficial effects on endophytic communities. Optimal concentrations of HS should be determined based on specific crop and environmental conditions to ensure that the right balance is achieved. Additionally, combining HS applications with bacterial inoculants like *Frigoribacterium* and *Hymenobacter* could further enhance plant resilience under stress.

Future research should explore the role of plants in the shifts observed in endophytic bacterial communities following the application of HS and PEG. This could involve creating synthetic microbial communities based on metabarcoding results to directly test the effects of HS and PEG on bacterial strains and communities as well as using RNAseq to identify the most expressed bacterial genes under different conditions. Additionally, the current study is limited by its 3-week duration, which may not have allowed the full capture of the long-term effects of HS and osmotic stress on plant growth and endophytic communities, particularly beyond the vegetative stage. Future studies should consider extending the experimental period to observe potential impacts throughout the entire cultivation cycle.

Moreover, a promising avenue for future research involves exploring how HS induce shifts in endophytic bacterial communities, guided by several key hypotheses. One hypothesis suggests that HS may favor bacterial species capable of using HS as electron donors in the anammox process. This could be tested by creating knockout mutants of specific genes involved in this pathway, such as hydrazine synthase (*hzsA*, *hzsB*, and *hzsC*) and hydrazine dehydrogenase (*hdh*) (Wang et al., 2016; Kuenen, 2008). Another hypothesis posits that HS form complexes with iron, reducing its availability and potentially increasing the presence of Actinobacteria (Xu et al., 2021). Testing this could involve reducing iron levels in hydroponic solutions instead of applying HS to see if the same effects are observed. Additionally, it is also hypothesized that HS might induce mild stress in plants, leading to shifts in bacterial communities. This could be investigated using tomato mutants lacking the *rop9* gene, which regulates ROS formation (Puli et al., 2024). These lines of inquiry could provide valuable insights into the mechanisms behind HS-induced microbial shifts and their impact on plant health and stress tolerance. Finally, studying different tomato cultivars, particularly those with varying stress tolerances and responses to HS, could reveal whether the microbial shifts and beneficial effects of HS are consistent across genetic backgrounds.

## Data Availability

The datasets presented in this study can be found in online repositories. The names of the repository/repositories and accession number(s) can be found in the article/**Supplementary Material**.
