# Supplementary material for: Humic substances increase tomato tolerance to osmotic stress while modulating vertically transmitted endophytic bacterial communities
Source: Front Plant Sci. 2024 Nov 19;15:1488671. doi: 10.3389/fpls.2024.1488671 (PMC11611569; doi:10.3389/fpls.2024.1488671)
Supplement: Supplementary file 1 [file DataSheet1.pdf]

Supplementary Material

1 SUPPLEMENTARY DATA

Raw data of the three experiment on the effect of humic substances on tomato growth are available on Zenodo: 10.5281/zenodo.13881235. The result of the metabarcoding study can be found on NCBI, datasets PRJNA1145306 (https://www.ncbi.nlm.nih.gov/bioproject/1145306).

2 SUPPLEMENTARY TABLES AND FIGURES

2.1 Tables

Table S1. Comparison of the osmotic potential of hydroponic solutions of the four treatments

**Comparison treatments**	**Sum Sq**	**Df**	**F value**	**Pr** $(> F)$)
Control vs HS	4.429e-06	1	0.25	0.6433
PEG vs PEG + HS	0.0003662	1	3.4543	0.1124

Table S2. Chemical characteristics of HS extracted from leonardite
**Dissolved organic carbon*

Parameter	Unit	Leonardite (HHS)
pH	-	10.2
EC	ms/cm	9
Total N	$mg.L^{-1}$	80
NH_4^+	$mg.L^{-1}$	20
Cl^-	$mg.L^{-1}$	$<$10
DOC*	$mg.L^{-1}$	18770
Fe	$mg.L^{-1}$	50
Co	$mg.L^{-1}$	0.022
Cr	$mg.L^{-1}$	0.059
Cu	$mg.L^{-1}$	0.091
Ni	$mg.L^{-1}$	0.049
Pb	$mg.L^{-1}$	0.2
Zn	$mg.L^{-1}$	0.114
As	$mg.L^{-1}$	0.0185
Cd	$mg.L^{-1}$	0.0011
Hg	$mg.L^{-1}$	0.00129

Table S3. Comparison of the efficiency of the photosystem II of tomato under non stress "Control" and osmotic stress "PEG" conditions.

Data from the three experiments were pooled and significant differences among treatments were assessed with Student's t-test ($p \leq 0.05$).

Parameters	Estimate	P value	Significance
Fv/Fm	0.009972	0.8617	-
PSII	-0.01147	0.9918	-

2.2 Figures

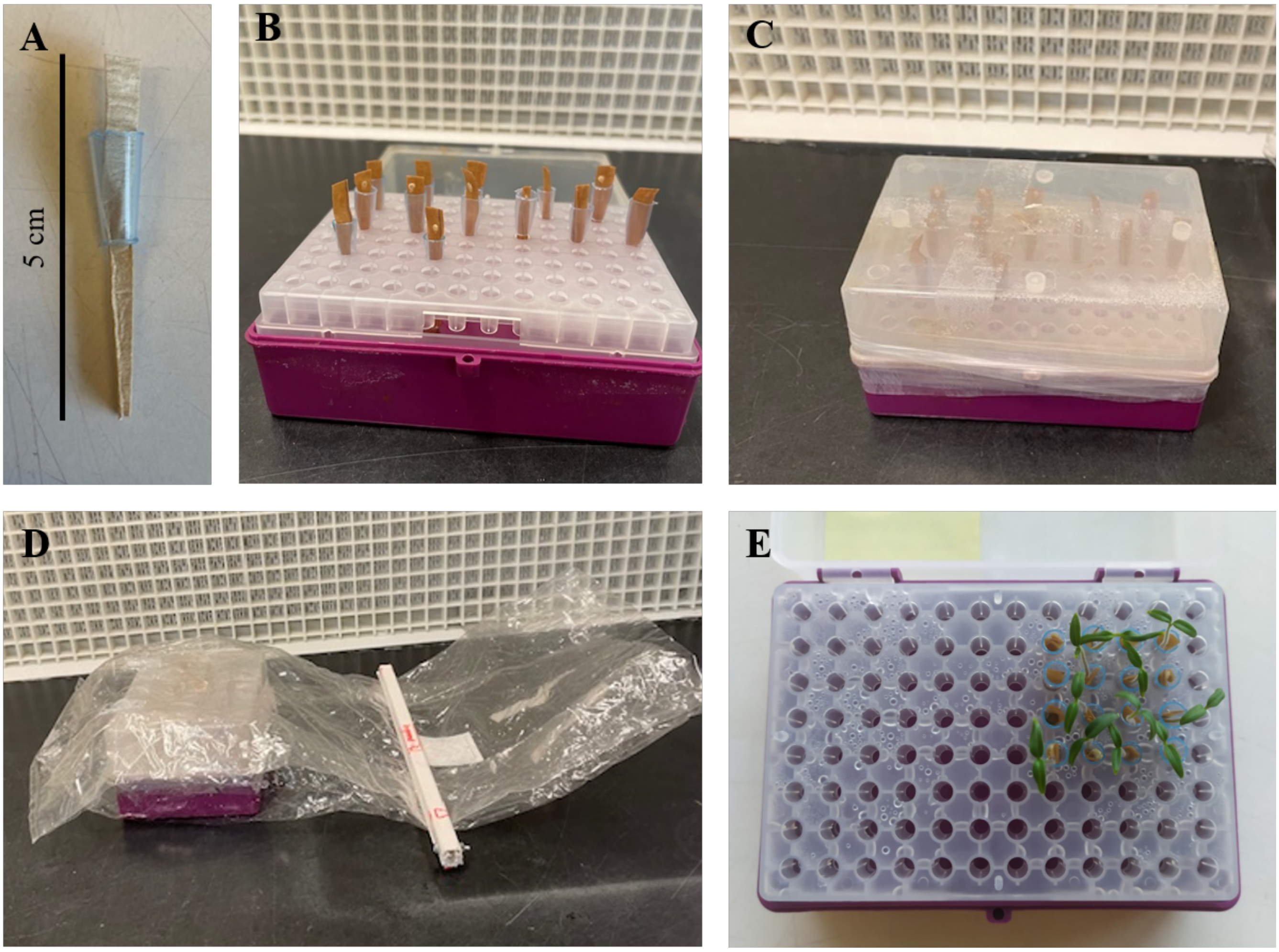

Figure S1. Sterile germination system – Cut tips containing absorbent paper (A) are placed in a tip box, and the entire setup is autoclaved. Under a laminar flow hood, the tips box is then filled with sterilized demineralized water, and surface-disinfected seeds are placed on the absorbent paper (B). The system is sealed with Parafilm (C) and enclosed in a sterile bag (D). Seeds are germinated for ten days, with seven days in the dark followed by three days in the light (E).

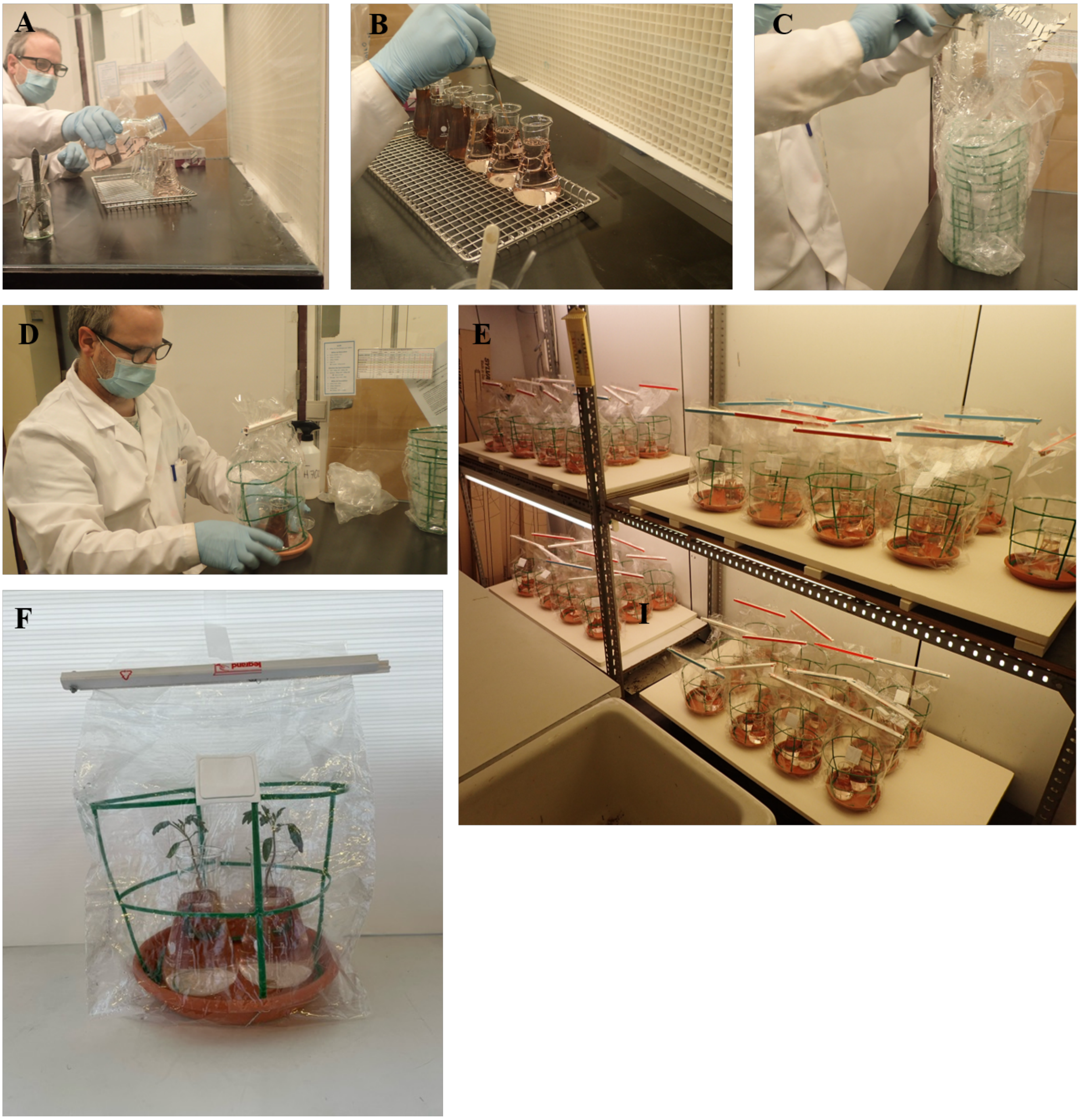

Figure S2. Sterile culture system – Erlenmeyer flasks were autoclaved inside an autoclavable bag and then filled with sterilized hydroponic solution under a laminar flow hood (A). Germinated seeds in tips were carefully placed inside the Erlenmeyer using a hook. The plastic frame and plant holder were also sterilized in autoclavable bags (C). The plastic frame was positioned on the plant holder, and the entire system was enclosed in an autoclavable bag with a 0.22 um filter (D). The Erlenmeyer, now inside the sterile bag, were transferred to a growth chamber for three weeks (E – F).

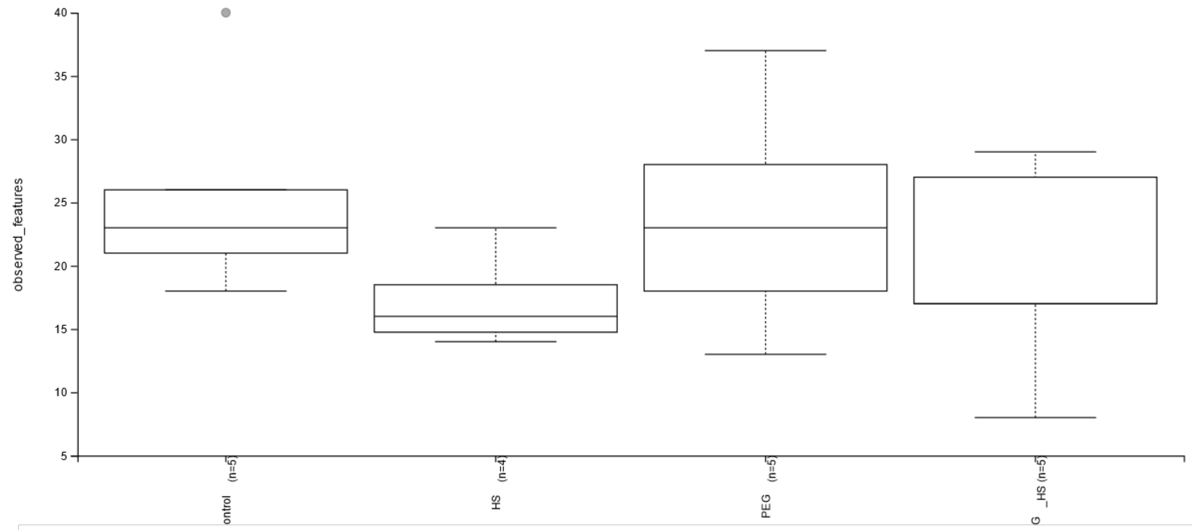

Kruskal-Wallis (all groups)

	Result
**H**	3.3787798408488143
**p-value**	0.33682760383840155

Kruskal-Wallis (pairwise)

Download CSV

Group 1	Group 2	H	p-value	q-value
**Control (n=5)**	**HS (n=4)**	3.403361	0.065064	0.390382
	**PEG (n=5)**	0.099387	0.752567	0.752567
	**PEG_HS (n=5)**	0.889024	0.345742	0.556013
**HS (n=4)**	**PEG (n=5)**	1.225210	0.268341	0.556013
	**PEG_HS (n=5)**	0.558621	0.454817	0.556013
**PEG (n=5)**	**PEG_HS (n=5)**	0.537805	0.463344	0.556013

Figure S3. Alpha diversity (Observed features) between bacterial endophytic communities of *S. lycopersicum* in tomato roots according to the treatment applied.
Comparison between tomato roots treated with humic substances (HS), under osmotic stress (PEG), under osmotic stress and treated with HS and control plants (PEG-HS).

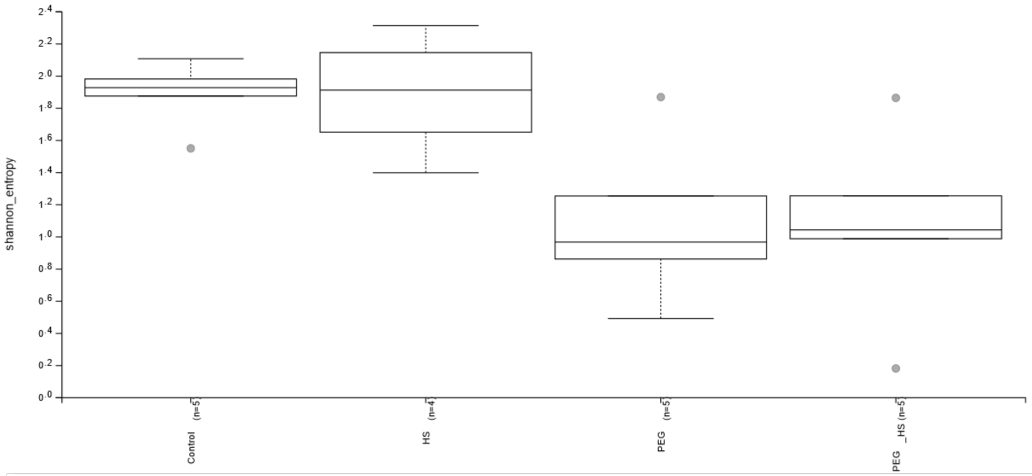

Kruskal-Wallis (all groups)

	Result
**H**	10.193684210526328
**p-value**	0.016989504405801603

Kruskal-Wallis (pairwise)

Download CSV

Group 1	Group 2	H	p-value	q-value
**Control (n=5)**	**HS (n=4)**	0.000000	1.000000	1.000000
	**PEG (n=5)**	5.770909	0.016294	0.048881
	**PEG_HS (n=5)**	5.770909	0.016294	0.048881
**HS (n=4)**	**PEG (n=5)**	3.840000	0.050044	0.075065
	**PEG_HS (n=5)**	3.840000	0.050044	0.075065
**PEG (n=5)**	**PEG_HS (n=5)**	0.098182	0.754023	0.904827

Braplot.pptx - PowerPoint

Figure S4. Alpha diversity (Shannon entropy) between bacterial endophytic communities of *S. lycopersicum* in tomato roots according to the treatment applied.
Comparison between tomato roots treated with humic substances (HS), under osmotic stress (PEG), under osmotic stress and treated with HS and control plants (PEG-HS).(C) Pielou eveness.

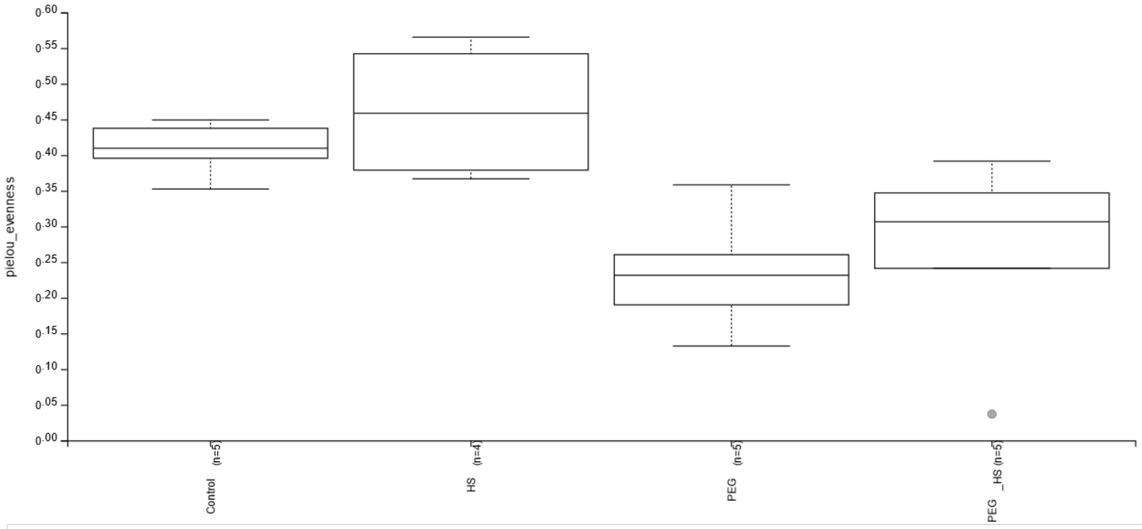

Kruskal-Wallis (all groups)

	Result
**H**	11.507368421052632
**p-value**	0.009276122597187896

Kruskal-Wallis (pairwise)

Download CSV

Group 1	Group 2	H	p-value	q-value
**Control (n=5)**	**HS (n=4)**	0.240000	0.624206	0.624206
	**PEG (n=5)**	5.770909	0.016294	0.032587
	**PEG_HS (n=5)**	5.770909	0.016294	0.032587
**HS (n=4)**	**PEG (n=5)**	6.000000	0.014306	0.032587
	**PEG_HS (n=5)**	3.840000	0.050044	0.075065
**PEG (n=5)**	**PEG_HS (n=5)**	0.534545	0.464702	0.557643

Figure S5. Alpha diversity (Pielou eveness) between bacterial endophytic communities of *S. lycopersicum* in tomato roots according to the treatment applied.
Comparison between tomato roots treated with humic substances (HS), under osmotic stress (PEG), under osmotic stress and treated with HS and control plants (PEG-HS).

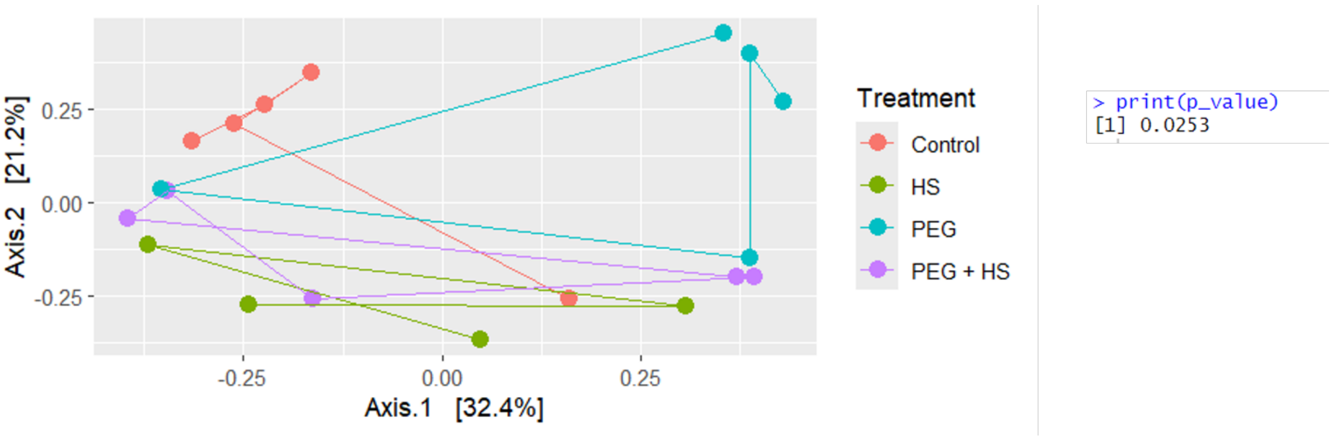

Figure S6. PCoA plot with sample distances inferred from bacterial endophytic community composition in roots of plants according to the treatment applied.
Plants treated with humic substances (HS), under osmotic stress (PEG), under osmotic stress and treated with HS and control plants (PEG-HS).

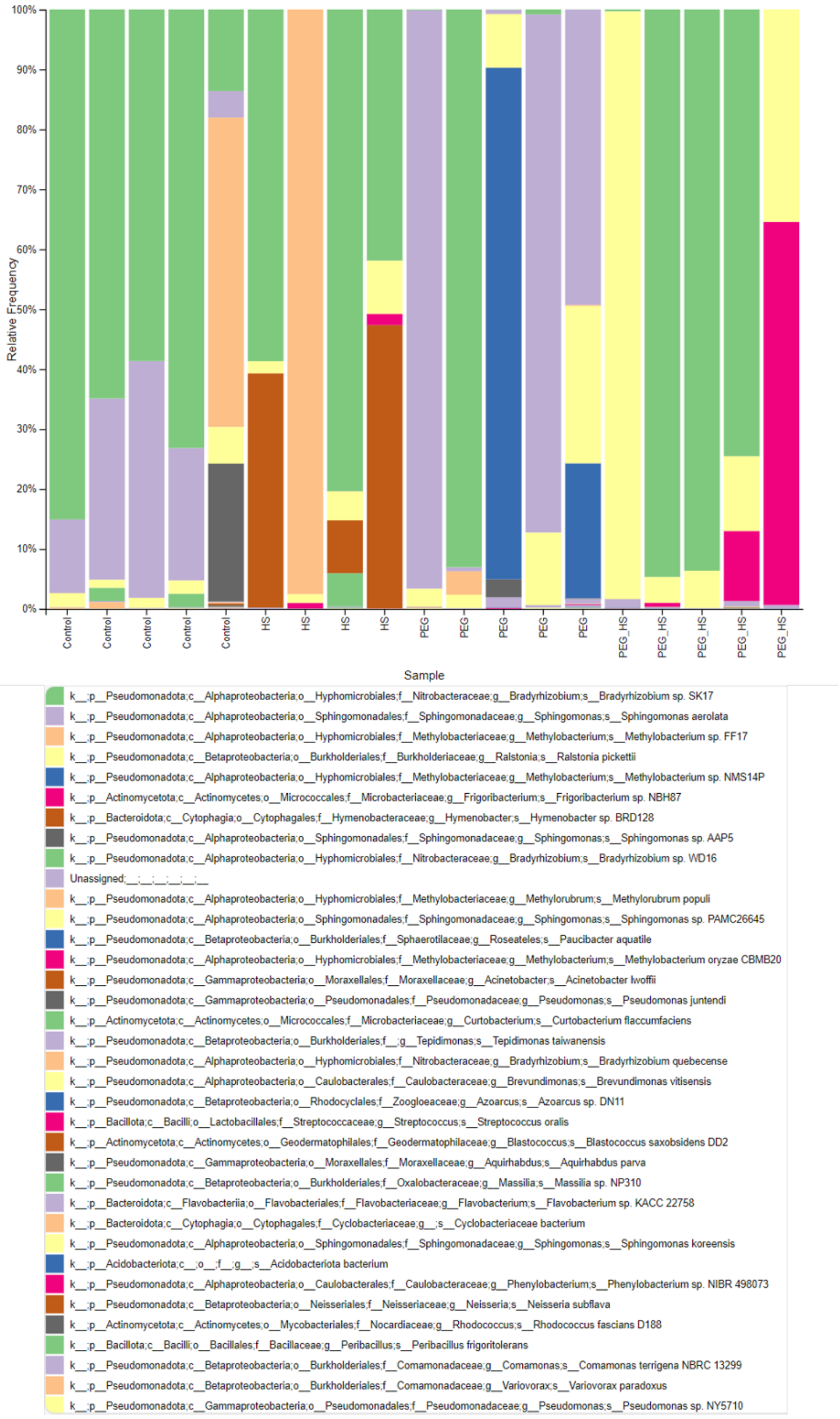

Figure S7. Relative abundance of endophytic bacterial taxa in the roots of tomato at genus level in each sample.
Control, HS, PEG-HS and PEG stand for plants under non stress, non stress and treated with humic substances (HS), osmotic stress and treated with HS and osmotic stress alone.
